# Uncovering Hidden Genetic Contributors to 46,XY Disorders of Sex Development Through Phenotype-Driven Rare Variant Assessment: A Pilot Study

**DOI:** 10.3390/genes17070798

**Published:** 2026-07-13

**Authors:** Yijun Tang, Yao Chen, Qianwen Zhang, Jie Tang, Yu Ding, Juan Li, Tingting Yu, Xiumin Wang

**Affiliations:** 1 Department of Endocrinology and Metabolism, Shanghai Children’s Medical Center, School of Medicine, Shanghai Jiao Tong University, Shanghai 200127, China; tangyijun@scmc.com.cn (Y.T.); chen-yao@scmc.com.cn (Y.C.); zhangqianwen@scmc.com.cn (Q.Z.); dingyu@scmc.com.cn (Y.D.); lijuan@scmc.com.cn (J.L.); 2 Department of Medical Genetics and Molecular Diagnostic Laboratory, Shanghai Children’s Medical Center, School of Medicine, Shanghai Jiao Tong University, Shanghai 200127, China; tangjie@scmc.com.cn

**Keywords:** rare variant assessment, 46,XY disorder of sex development, bioinformatics analysis, SKAT-O, GO enrichment

## Abstract

Background: Despite advances in genetic testing, many 46,XY Disorders of sex development (DSD) cases remain unsolved after whole-exome sequencing (WES). This study intended to explore rare variants in patients with micropenis, cryptorchidism, or hypospadias using bioinformatics analysis to identify potential pathogenic contributors and pathways underlying 46,XY DSD. Methods: A total of 35 patients with specific phenotypes (micropenis/cryptorchidism/hypospadias) and negative whole-exome sequencing results were enrolled. Bioinformatics analysis methods (SKAT-O test and GO enrichment) were applied to identify the putative loss-of-function (pLoF) variation, including nonsense, frameshift, and canonical splice-site variants, and predicted deleterious missense variants (CADD Phred > 20). Literature was reviewed to explore the correlation of detected candidate genes/pathways and 46,XY disorder of sex development. Results: After variant quality filtering, we identified 307,638 pLoF variants and 127,857 predicted deleterious missense variants across all samples. In subgroup A (micropenis, *n* = 21), we identified 146,268 pLoF variants and 104,746 predicted deleterious missense variants. In subgroup B (cryptorchidism, *n* = 10), we identified 111,172 pLoF variants and 77,244 predicted deleterious missense variants. In subgroup C (hypospadias, *n* = 4), we identified 50,198 pLoF variants and 23,111 predicted deleterious missense variants. Using SKAT-O with an initial screening threshold of *p* < 0.005 (FDR q < 0.05), we obtained 67 candidate genes from the pLoF variant set and 59 candidate genes from the predicted deleterious missense variant set in subgroup A; 81 and 11 candidate genes, respectively, in subgroup B; and 17 and 0 candidate genes, respectively, in subgroup C. Conclusions: Assessment of rare variants helps further explore the genetic contributors to 46,XY disorder of sex development and provide potential candidate genes and associated pathways.

## 1. Introduction

Disorders of sex development (DSD) are a group of congenital conditions characterized by discordance among chromosomal karyotype, gonadal phenotype, and anatomical development of the gonads [1]. The reported incidence of DSD is estimated to be approximately 1/4500 live births [1]. Based on karyotype, DSD patients can be broadly classified into three categories: sex chromosome DSD, 46,XY DSD, and 46,XX DSD [2].

Patients with 46,XY DSD exhibit significant clinical heterogeneity across different disease subtypes and age groups [3]. Their clinical manifestations vary widely in severity, including micropenis, cryptorchidism, hypospadias, inguinal hernia, and the absence of secondary sexual characteristics (e.g., lack of breast development, primary amenorrhea, poor testicular development, and micropenis) [1,2,3]. The pathogenesis of 46,XY DSD is still under active investigation. In our previous work using targeted sequencing and whole-exome sequencing in a cohort of Chinese 46, XY DSD patients, we have identified causative or candidate variants in a broad range of genes, including: Anti-Müllerian hormone (*AMH*), Anosmin 1 (*ANOS1*), Androgen receptor (*AR*), Chromodomain helicase DNA binding protein 7 (*CHD7*), Cytochrome P450 family 17 subfamily A member 1 (*CYP17A1*), Desert hedgehog signaling molecule (*DHH*), Fibroblast growth factor receptor 1 (*FGFR1*), Follicle stimulating hormone receptor (*FSHR*), Growth hormone receptor (*GHR*), GLI family zinc finger 2 (*GLI2*), Gonadotropin releasing hormone receptor (*GNRHR*), Hydroxysteroid 17-beta dehydrogenase 3 (*HSD17B3*), KISS1 receptor (*KISS1R*), Luteinizing hormone/choriogonadotropin receptor (*LHCGR*), Mitogen-activated protein kinase kinase kinase 1 (*MAP3K1*), Notch receptor 3 (*NOTCH3*), Nuclear receptor subfamily 0 group B member 1 (*NR0B1*), Nuclear receptor subfamily 5 group A member 1 (*NR5A1*), Pleckstrin homology domain interacting protein (*PHIP*), Cytochrome P450 oxidoreductase (*POR*), Prokineticin receptor 2 (*PROKR2*), Protein tyrosine phosphatase non-receptor type 11 (*PTPN11*), Ras-like without CAAX 1 (*RIT1*), SRY-box transcription factor 10 (*SOX10*), SRY-box transcription factor 2 (*SOX2*), SRY-box transcription factor 3 (*SOX3*), Steroid 5-alpha-reductase 2 (*SRD5A2*), Sex determining region Y (*SRY*), Tachykinin receptor 3 (*TACR3*), T-box transcription factor 3 (*TBX3*), TSPY-like 1 (*TSPYL1*), Zinc finger protein, FOG family member 2 (*ZFPM2*) [4].

Despite the widespread use of whole-exome sequencing (WES) in clinical genetics, a significant proportion of 46,XY DSD patients remain without a definitive molecular diagnosis, with approximately 50% classified as WES-negative [4,5]. This persistent diagnostic gap underscores the limitations of conventional approaches focused on monogenic or coding-region variants, particularly for conditions like DSD, where phenotypic heterogeneity, incomplete penetrance, and polygenic or oligogenic inheritance may contribute to pathogenesis [6]. The inability to resolve the genetic basis of these cases not only hampers personalized clinical management but also obscures our understanding of the molecular mechanisms governing sex differentiation.

Recent advances in bioinformatics and rare variant assessment studies have highlighted the potential role of aggregated rare variants in complex developmental disorders [7]. However, their contribution to 46,XY DSD remains under-explored, especially in WES-negative cohorts. Rare variants acting synergistically within biological pathways or non-coding regulatory elements may underlie unresolved phenotypes [8].

In this study, we aimed to address these gaps by integrating rare variant burden testing with pathway enrichment analysis to explore the genetic architecture of 46,XY DSD in WES-negative patients. We focused on three clinically distinct phenotypes (micropenis, cryptorchidism, and hypospadias) to evaluate whether these phenotypes arise from divergent molecular pathways or shared developmental disruptions. By applying the SKAT-O method, a robust statistical framework for detecting rare variant associations across genes or gene sets, we sought to overcome the power limitations of single-variant analyses and identify candidate genes harboring aggregated rare variants. Subsequent pathway enrichment analysis was employed to map these genes onto biological networks, thereby uncovering potential mechanistic links to sex development.

## 2. Materials and Methods

### 2.1. Participants

This investigation encompassed a cohort of patients diagnosed with 46,XY DSD and exhibited negative outcomes from WES analyses at Shanghai Children’s Medical Center (SCMC) from 2016 to 2023.

Micropenis, cryptorchidism, and hypospadias were selected because they represent the most frequent phenotypic presentations of 46,XY DSD with negative WES results in our previous work [4]. To ensure a phenotype-driven approach, only patients with one of these three phenotypes as the primary clinical feature were included; individuals with complex syndromic DSD or ambiguous genitalia without a clear predominant phenotype were excluded. Importantly, all enrolled patients had undergone comprehensive clinical evaluation and were confirmed to have a diagnosis of 46,XY DSD based on the presence of a male karyotype (46,XY) together with atypical gonadal or anatomical development.

A total of 35 subjects (21 presented with micropenis, 10 with cryptorchidism, and 4 with hypospadias) were eventually included. Based on different clinical phenotypes, the subjects were divided into different subgroups: patients phenotyped as micropenis were divided into subgroup A, patients phenotyped as cryptorchidism were divided into subgroup B, and patients phenotyped as hypospadias were divided into subgroup C. These three phenotypes were analyzed separately to explore whether distinct molecular pathways underlie each clinical entity. Clinical data, including medical history, physical examination, and paraclinical examinations, were collected and evaluated by clinicians.

In addition, the current study enrolled 643 healthy adult males without any pubertal development issues as controls. The genomic data of controls were used as a reference control group and were then compared with the genomic data of study subjects to screen for differential variant information. The inclusion and collection of genomic data in the control group were from previously published work [9].

### 2.2. Genomic Data Processing

All 35 pediatric 46,XY DSD cases and 643 healthy male controls were processed using identical laboratory and analytical procedures. All original genomic data were obtained with written informed consent from the guardians of the patients (below 18 years old) or from the control group subjects themselves (643 healthy male adults) at the time of collection, consenting to the use of their genomic data for scientific research and publication. The study was approved by the Ethics Committee of SCMC, Shanghai Jiao Tong University School of Medicine (No. SCMCIRB-K2016013).

All participants were of Chinese Han ancestry and were recruited from east China. Genomic DNA was extracted from peripheral blood samples of all 35 pediatric DSD cases and 643 healthy controls using standard procedures. DNA was extracted from blood samples of subjects. Sequencing libraries were prepared using the SureSelect Human All Exon V6 enrichment kit (Agilent, Santa Clara, CA, USA) following the manufacturer’s protocol, which included DNA fragmentation, adapter ligation, hybridization, amplification, and purification. Paired-end sequencing (2 × 100 bp) was performed on the HiSeq 2500 platform (Illumina, Inc., San Diego, CA, USA). The average sequencing depth exceeded 100× for all samples, and the same quality control filters were applied uniformly across both groups.

Raw sequencing reads were assessed for quality using FastQC (version 0.11.9) and Fastp (version 0.20.1) for adapter trimming and low-quality read filtering. Clean reads were aligned to the human reference genome (GRCh37/hg19) using Speedseq (version 0.1.2). Alignment quality was evaluated using mosdepth (version 0.3.1) and bamdust (version 1.0.9), with metrics including mapping rate, PCR duplicate rate, average sequencing depth, and coverage uniformity. Variant discovery and genotyping were performed using the Genome Analysis Toolkit (GATK4, version 4.2.0.0) following the Best Practices workflow [10]. Importantly, cases and controls were genotyped simultaneously using GATK joint calling via the GenomicsDB module, which minimizes technical batch effects. Variant quality score recalibration (VQSR) was applied to filter variants and achieve the desired accuracy and sensitivity. Our burden analysis included only variants located within regions with adequate coverage (depth ≥ 10×) across all samples, ensuring that no systematic coverage bias between cases and controls affected variant counts. Copy number variants (CNVs) were identified using CNVkit (version 0.9.8).

The variant call format files of all patients and controls were merged using bcftools (version 1.12). Annotation was performed using ANNOVAR software (8 June 2020 edition), referencing gnomAD, mutant allele fractions (MAFs), and combined annotation dependent depletion (CADD) predicted scores. Rare variants were defined as those with a minor allele frequency (MAF) < 0.01% in the gnomAD database. Based on the annotation, variants were filtered to include: (1) putative loss-of-function(pLoF) variants, including stop-gain, start-loss, and splice site variants; (2) predicted deleterious missense variants, including missense variants with CADD scores showing phred > 20. Variants were aggregated at the gene level for analysis.

### 2.3. SKAT-O Test

The SKAT-O (Optimal sequence kernel association test) test for pLoF and predicted deleterious missense variants was implemented using the SKAT package in R Studio [11]. SKAT-O combines SKAT with burden test statistics by leveraging correlations to transform multiple genetic variant information into a single genetic score, then performing association analysis between the score and phenotype [11]. It utilizes efficient one-dimensional numerical integration to compute approximate *p*-values for SKAT-O [12]. Therefore, it is suitable for two common analysis scenarios: (1) when there are multiple non-associated variants or variants with inconsistent associations within the same analyzed region; (2) when there are multiple associated variants with the same association direction within the same region [9].

This method aggregates single nucleotide polymorphism (SNP) score statistics to effectively calculate the overall SNP *p*-value. A gene was considered to show a significant aggregate association if the nominal *p*-value was less than 0.005. To account for multiple testing across genes, we applied the Benjamini-Hochberg false discovery rate (FDR) procedure, and genes with an FDR-adjusted q-value < 0.05 were retained as candidate genes.

### 2.4. Pathway Enrichment Analysis

For genes marked as significantly different by SKAT-O testing, the Cluster Profiler R package was used for Gene Ontology (GO) enrichment analysis, Kyoto Encyclopedia of Genes and Genomes (KEGG) enrichment analysis, and Reactome pathway association analysis [9]. The background gene set consisted of all protein-coding genes annotated in the respective database that passed the initial quality control filters of our whole-exome sequencing data.

For each pathway or GO term, the significance of enrichment was assessed using a one-sided Fisher’s exact test. This test computes the probability of observing the number of candidate genes in a given pathway under the null hypothesis of no enrichment. To account for multiple comparisons across all tested pathways, *p* values were adjusted using the Benjamini-Hochberg FDR procedure. A pathway or GO term was considered significantly enriched if the adjusted *p*-value was less than 0.05. Enriched terms meeting this threshold were retained for subsequent interpretation.

### 2.5. Targeted Narrative Review

To contextualize our genetic findings, we performed a targeted narrative review focusing on the candidate genes identified in our SKAT-O analysis. For each candidate gene, we conducted comprehensive searches across multiple databases, including PubMed, OMIM, and NCBI Gene resources. Searches were performed using combinations of the gene name and DSD-related phenotype terms, such as “disorders of sex development,” “micropenis,” “cryptorchidism,” “hypospadias,” and related synonyms. Articles and entries were manually screened for relevance, and we specifically assessed whether the gene had been previously reported in association with 46,XY DSD or the corresponding phenotypic subgroups.

## 3. Results

### 3.1. Clinical and Demographic Characteristics of Participants

**Figure 1:**
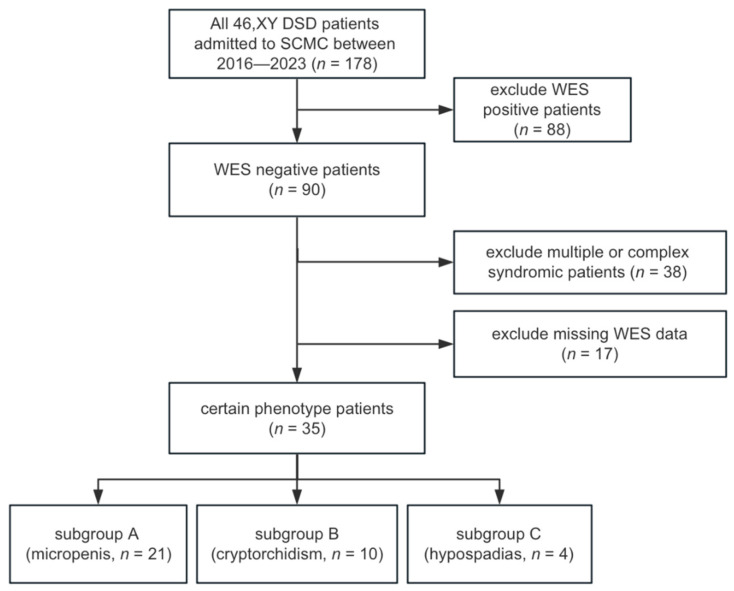
Participants flowchart of the study. DSD, disorders of sex development; SCMC, Shanghai Children’s Medical Center; WES, whole-exome sequencing.

A total of 35 subjects from the SCMC department of endocrinology and metabolism between 2016 and 2023 were enrolled, including 21 subjects phenotyped as micropenis in subgroup A, 10 phenotyped as cryptorchidism in subgroup B, and 4 phenotyped as hypospadias in subgroup C (Figure 1). All subjects were clinically diagnosed as 46,XY DSD and exhibited negative outcomes from WES analyses.

The median diagnostic age was 9.00 (3.17, 12.25) years old in subgroup A, 8.50 (3.21, 13.29) years old in the cryptorchidism group, and 3.00 (0.98, 6.65) years old in the hypospadias group. The median age of patients in subgroup C was lower than that of the other two subgroups, likely because hypospadias is typically identified at birth due to its visible anatomical abnormality, whereas micropenis and cryptorchidism may be noticed later.

Basal hormone levels of LH, FSH, and testosterone were assessed to determine the function of the hypothalamic-pituitary-gonadal axis in all subjects at the time of diagnosis. According to the results, hormone levels are similar between subgroups; however, differences exist within each subgroup. Most subjects manifested low FSH and LH levels. However, there was one subject in subgroup A who showed elevated FSH (10.14 mIU/mL) and LH (5.75 mIU/mL) levels, and one subject in subgroup B who showed elevated FSH (47.09 mIU/mL) and elevated LH (4.72 mIU/mL) levels. Laboratory parameter results are summarized in Table 1. Subgroup C comprised only four individuals; thus, its clinical and endocrine characteristics were not summarized separately.

### 3.2. Rare Variant Screening and SKAT-O Test

After GATK variant analysis and annotation of WES data, and classification of rare variants based on different types and CADD Phred > 20, a total of 307,638 pLoF variants and 127,857 predicted deleterious missense variants were identified. Among these, there were 146,268 pLoF variants and 104,746 predicted deleterious missense variants in subgroup A; 111,172 pLoF variants and 77,244 predicted deleterious missense variants in subgroup B; and 50,198 pLoF variants and 23,111 predicted deleterious missense variants in subgroup C. With 678 individuals (35 cases + 643 controls), all reported variant counts represent unique variant sites identified across the entire cohort after rigorous quality filtering.

Subsequently, SKAT-O statistics were calculated to determine whether rare variants were enriched in the assumed LOF or predicted deleterious missense variants mentioned above. Finally, after testing (*p* < 0.005, FDR q < 0.05), 67 candidate genes with pLoF variants and 59 candidate genes with predicted deleterious missense variants were obtained for the subgroup A, 81 candidate genes with pLoF variants and 11 candidate genes with predicted deleterious missense variants were obtained for the subgroup B, and 17 candidate genes with pLoF variants were obtained for the hypospadias subgroup, and no candidate genes with predicted deleterious missense variants were obtained likely due to the small sample size of this subgroup.

After comprehensive pathway enrichment analysis, the genes involved in the pathways were identified through literature searches to determine their association with the pathogenesis of disorders of sex development (see Table 2).

### 3.3. Micropenis—Subgroup A Pathway Enrichment

**Figure 2:**
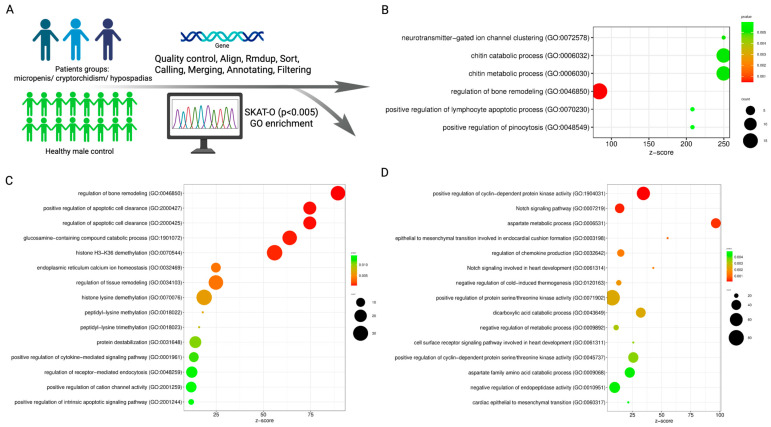
Scheme and enrichment results of rare variant SKAT-O assessment in micropenis/cryptorchidism/subgroup C versus control group. (**A**) Genetic data processing of rare variant assessment. (**B**) Pathway enrichment result in subgroup C. (**C**) Pathway enrichment result in subgroup B. (**D**) Pathway enrichment result in subgroup A.

Initially, GO enrichment analysis was performed for the 105 differential genes in the subgroup A. After visualizing the top 15 enriched signaling pathways of the micropenis group GO enrichment results using the Cluster Profiler R package, the results of pathway enrichment, including gene variation numbers, pathway network diagrams, and pathway correlations, are shown in the figures and tables below (see Figure 2D). All enriched signaling pathways involved various processes such as positive regulation of cyclin-dependent protein kinase activity, the Notch signaling pathway, as well as metabolic and developmental processes. It can be observed that pathways related to the positive regulation of cyclin-dependent protein kinase activity had higher correlation coefficients, and pathways involving the positive regulation of serine/threonine kinase activity detected a higher number of rare variants.

### 3.4. Cryptorchidism—Subgroup B Pathway Enrichment

After performing GO enrichment analysis for the 91 differential genes in subgroup B, results involved pathways on regulation of skeletal remodeling, regulation of apoptotic cell clearance, metabolic processes involving glucosamine compounds, histone H3-K36 demethylation, regulation of endoplasmic reticulum calcium ion homeostasis, histone lysine demethylation, peptide lysine methylation and trimethylation, protein stability, signaling pathways mediated by cytokines, regulation of receptor-mediated endocytosis, positive regulation of cation channel activity, and positive regulation of apoptosis signaling pathways. (see in Figure 2C).

### 3.5. Hypospadias—Subgroup C Pathway Enrichment

After performing GO enrichment analysis for the 17 differential genes in the hypospadias group, the network diagrams of all enriched signaling pathways are shown below (see Figure 2B). These pathways include processes such as chitin decomposition metabolism, neurotransmitter-gated ion channels, regulation of phagocytosis, positive regulation of lymphocyte apoptosis, regulation of skeletal remodeling, protein palmitoylation, cellular response to interferon, lysosome acidification, metabolic processes involving glucosamine compounds, positive regulation of lipase activity, and histone H3-K36 demethylation. It can be observed that pathways related to neurotransmitter-gated ion channels had higher correlation coefficients, and a higher number of rare variants were detected in pathways involving the regulation of skeletal remodeling.

## 4. Discussion

The diagnostic age differs across subgroups—older in subgroup A versus subgroup C—may reflect varying thresholds for clinical intervention. For instance, micropenis often becomes apparent during mini-puberty or puberty, whereas hypospadias is typically identified at birth [13]. These age-related dynamics emphasize the need for longitudinal studies to assess how genetic variants influence phenotype expressivity over time [14].

The use of rare variant assessment in conjunction with the SKAT-O test allowed us to explore numerous potential pLoF and predicted deleterious missense variants emerging from the analysis [15]. Candidate genes potentially associated with the phenotype of sex development abnormalities were screened out among all variants. Most of these genes have only been reported in individual cases. The prioritization of ADAM17, PKD2, RBPJ, and LEPR highlights the convergence of diverse pathways on common phenotypic outcomes. For example, ADAM17, a sheddase regulating Notch pathway activation, may indirectly influence urogenital development by modulating extracellular matrix components or growth factor bioavailability [16]. The protein encoded by RBPJ is a transcriptional regulator important in the Notch signaling pathway, acting as a repressor when not bound to Notch proteins and an activator when bound to Notch proteins [17]. Another gene identified in this study, PKD2, is one of the classical pathogenic genes for renal and reproductive ductal development, aligning with its putative role in epididymal coiling defects, as seen in murine models [18]. Disruption of PKD2 may lead to dilation of the proximal renal tubules/collecting ducts, resulting in failed epididymal coiling and presenting as testicular dysgenesis [19]. It has also been found that loss of Pkd2 in epithelial cells leads to reproductive tract defects [20]. The link between LEPR and hypogonadotropic hypogonadism provides a mechanistic bridge between metabolic and reproductive systems. Leptin receptor dysfunction may disrupt GnRH pulsatility, exacerbating hormone deficiencies [21]. Emerging evidence also links LEPR deficiency to gonadal dysfunction and hypogonadotropic hypogonadism, providing a potential molecular explanation for obesity-related reproductive anomalies in DSD [22].

The pathway enrichment patterns observed across the three subgroups underscore the importance of pathogenesis stratification in DSD research. For instance, the subgroup A exhibited associations with cyclin-dependent kinase activity and the Notch signaling pathway. Cyclin-dependent kinases are critical regulators of cell cycle progression, and their dysregulation has been implicated in gonadal hypoplasia and diminished cell proliferation, which could affect testosterone synthesis and penile growth [23]. The Notch pathway, known for its role in cell fate determination and endocrine organ development, may further contribute to male reproductive tract development and function [24]. This aligns with the observed hormonal profile in this group, including low median testosterone and LH levels, suggesting a potential interplay between genetic defects and hypothalamic-pituitary-gonadal axis dysfunction.

In contrast, subgroup B showed enrichment for pathways such as histone lysine demethylation and regulation of bone remodeling. Histone modification processes are increasingly recognized for their role in testicular descent, as epigenetic regulation of genes like INSL3 and RXFP2 is essential for gubernaculum development [25]. INSL3, by finely tuning bone formation and resorption, is also involved in bone remodeling processes [26]. These findings raise the possibility that cryptorchidism in this cohort arises from defects in epigenetic programming, rather than isolated hormonal deficiencies.

The subgroup C, though small in sample size, uniquely implicated neurotransmitter-gated ion channels, which have been linked to urethral plate fusion in animal models [27]. The FOXA1 transcription factor network may orchestrate development of the urethral tube through an autoregulatory loop with Shh signaling [28].

Overall, the absence of clear phenotypic associations for some of the candidate genes suggests that the pathogenesis of 46,XY DSD is likely oligogenic, with multiple genetic factors interacting in complex ways to influence disease development [29].

In summary, this exploratory study identifies several candidate genes potentially associated with 46,XY DSD in a Chinese pediatric cohort. Owing to the small sample size, lack of functional validation, and absence of independent replication, these findings should be considered hypothesis-generating rather than definitive. We acknowledge that we did not perform formal population stratification analysis (e.g., PCA), and that cases and controls were sequenced in different batches, although identical protocols and joint calling were applied to minimize technical batch effects. The observed genotype-phenotype correlations are preliminary and require validation in larger, well-phenotyped cohorts. Functional studies are needed to assess the biological relevance of the identified genes, and clinical or therapeutic applications are premature at this stage.

## 5. Conclusions

In this exploratory study, we identified several candidate genes through rare-variant burden analysis in a cohort of 46,XY DSD patients with negative WES results. These findings provide a preliminary framework for understanding the potential polygenic or oligogenic contributions to DSD pathogenesis. Our study highlights the value of burden testing in WES-negative DSD patients while underscoring the need for further investigation to confirm the biological and clinical relevance of the candidate genes identified.

## Figures and Tables

**Table 1:** Clinical characteristics of subjects in subgroup A and subgroup B enrolled in this study.

Subgroup	Subgroup A (Micropenis, *n* = 21)	Subgroup B (Cryptorchidism, *n* = 10)
Age (years)	9.00 (3.17, 12.25)	8.50 (3.21, 13.29)
Height SDS	0.52 ± 1.19	−0.24 ± 1.32
Weight SDS	1.55 ± 1.48	0.88 ± 1.51
BMI SDS	0.81 ± 1.42	0.60 ± 1.45
Testicular volume (mL) via Prader orchidometer	2.0 (1.5, 2.5)	Not palpable
Penile length (cm)	2.4 (2.0, 2.9)	3.5 (2.0, 4.7)
Testosterone (ng/mL)	0.12 (0.11, 0.26)	0.12 (0.09, 0.18)
DHT (pg/mL)	123.01 (42.15, 335.90)	124.55 (83.88, 213.04)
FSH (mIU/mL)	1.83 (0.49, 4.05)	1.92 (0.49, 12.66)
LH (mIU/mL)	0.24 (0.17, 2.04)	0.20 (0.09, 3.11)
GnRH stimulation		
LH peak (uIU/mL)	1.86 (1.57, 7.01)	2.98 (1.46, 6.68)
FSH peak (uIU/mL)	7.51 (3.61, 11.60)	5.53 (1.54, 12.12)
HCG stimulation		
Testosterone after stimulation (ng/mL)	1.17 (0.82, 2.14)	1.24 (0.33, 2.14)
△ Testosterone (ng/mL)	1.01 (0.63, 1.93)	0.94 (0.16, 1.66)
Testosterone/DHT	0.85 (0.47, 1.34)	0.50 (0.17, 1.64)

△ Testosterone, the testosterone response to hCG was calculated as the change from baseline.; SDS, Standard deviation score; BMI, Body mass index; DHT, dihydrotestosterone; FSH, follicle-stimulating hormone; LH, Luteinizing hormone.

**Table 2:** Differential genes involved in the top 15 pathway enrichment in rare variant assessment of 46,XY DSD.

Gene ID	Name	Chr	Classification	Phenotype (OMIM)
Subgroup A (micropenis)				
*ADAM17*	ADAM metallopeptidase domain 17	2p25.1	predicted deleterious missense variant	Inflammatory skin and bowel disease, neonatal
*ASPA*	Aspartoacylase	17p13.3	predicted deleterious missense variant, pLoF	Canavan disease
*DDO*	D-aspartate oxidase	6q21	pLoF	NA
*DTX3L*	Deltex E3 ubiquitin ligase 3L	3q21.1	predicted deleterious missense variant, pLoF	NA
*IL7*	Interleukin 7	8q12.3	pLoF	Immunodeficiency 130 with HPV-related verrucosis
*LTF*	Lactotransferrin	3p21.31	predicted deleterious missense variant	NA
*NR1D1*	Nuclear receptor subfamily 1 group D member 1	17q11.2	predicted deleterious missense variant	NA
*PKD2*	Polycystin 2, transient receptor potential cation channel	4q22.1	predicted deleterious missense variant, pLoF	Polycystic kidney disease 2
*RBPJ*	Recombination signal binding protein for immunoglobulin kappa J region	4p15.2	predicted deleterious missense variant	Adams-Oliver syndrome 3
*SERPINB5*	serpin family B member 5	18q21.33	predicted deleterious missense variant	NA
*SLC27A1*	Solute carrier family 27 member 1	19p13.11	pLoF	NA
*SNAI2*	Snail family transcriptional repressor 2	8q11.21	predicted deleterious missense variant, pLoF	NA
*SPOCK1*	SPARC (osteonectin), cwcv and kazal like domains proteoglycan 1	5q31.2	predicted deleterious missense variant	NA
*STOX1*	Storkhead box 1	10q22.1	pLoF	Preeclampsia/eclampsia 4
Subgroup B (cryptorchidism)				
*AMDHD2*	Amidohydrolase domain containing 2	16q24.3	pLoF	NA
*ANTKMT*	Adenine nucleotide translocase lysine methyltransferase	3p21.31	pLoF	NA
*C3*	Complement C3	19p13.3	predicted deleterious missense variant	Hemolytic uremic syndrome, atypical; Macular degeneration, age-related; C3 deficiency
*CASQ1*	Calsequestrin 1	1q21.3	predicted deleterious missense variant	Myopathy, vacuolar, with CASQ1 aggregates
*CCDC47*	Coiled-coil domain containing 47	17q25.3	pLoF	Trichohepato neuro-developmental syndrome
*CD300LF*	CD300 molecule like family member f	17q25.3	pLoF	NA
*CHIT1*	Chitinase 1	1q32.1	pLoF	Chitotriosidase deficiency
*FIS1*	Fission, mitochondrial 1	7q22.1	pLoF	NA
*IL7*	Interleukin 7	8q12.3	pLoF	Immunodeficiency 130 with HPV-related verrucosis
*KDM4A*	Lysine demethylase 4A	1p34.1	predicted deleterious missense variant	NA
*KDM8*	Lysine demethylase 8	16q12.2	pLoF	NA
*LEPR*	Leptin receptor	1p31.3	pLoF	Obesity, morbid, due to leptin receptor deficiency
*PPT1*	Palmitoyl-protein thioesterase 1	1p34.2	predicted deleterious missense variant, pLoF	Ceroid lipofuscinosis, neuronal
*PYHIN1*	Pyrin and HIN domain family member 1	1q23.1	predicted deleterious missense variant	NA
*SFPQ*	Splicing factor proline and glutamine rich	1p34.3	pLoF	NA
*SUCO*	SUN domain-containing ossification factor	2q33.3	predicted deleterious missense variant	NA
Subgroup C (hypospadias)				
*ANO10*	Anoctamin 10	3p22.1	pLoF	Spinocerebellar ataxia
*C1orf87*	Chromosome 1 open reading frame 87	1q21.3	pLoF	NA
*CDCA7*	Cell division cycle associated 7	1p34.3	pLoF	Immunodeficiency-centromeric instability-facial anomalies syndrome 3
*CHIT1*	Chitinase 1	1q32.1	pLoF	Chitotriosidase deficiency
*GRIK5*	Glutamate ionotropic receptor kainate type subunit 5	19q13.33	pLoF	NA
*IL19*	Interleukin 19	1q32.1	pLoF	NA
*KDM4A*	Lysine demethylase 4A	1p34.1	pLoF	NA
*LHFPL4*	LHFPL tetraspan subfamily member 4	3p22.3	pLoF	NA
*PDCD1*	Programmed cell death 1	2q37.3	pLoF	Autoimmune disease, multisystem, infantile onset
*PLEKHA6*	Pleckstrin homology domain containing A6	12p12.3	pLoF	NA
*PPT1*	Palmitoyl-protein thioesterase 1	1p34.2	pLoF	Ceroid lipofuscinosis, neuronal
*PYHIN1*	Pyrin and HIN domain family member 1	1q23.1	pLoF	NA
*RHOC*	Ras homolog family member C	1q21.3	pLoF	NA
*SLC4A5*	Solute carrier family 4 member 5	2q36.1	pLoF	NA
*SNU13*	Small nuclear ribonucleoprotein 13	22q13.1	pLoF	NA
*SUCO*	SUN domain-containing ossification factor	2q33.3	pLoF	NA

NA, not available; pLoF, putative loss-of-function.

## Data Availability

The data presented here are available on request from the corresponding author. The data are not publicly available due to privacy and ethical issues.
